# Predictors of neurologic outcomes and mortality in physically abused and unintentionally injured children: a retrospective observation study

**DOI:** 10.1186/s40001-023-01430-x

**Published:** 2023-10-17

**Authors:** En-Pei Lee, Shao-Hsuan Hsia, Jainn-Jim Lin, Oi-Wa Chan, Han-Ping Wu

**Affiliations:** 1 Division of Pediatric Critical Care Medicine, Department of Pediatrics, Linko Chang Gung Memorial Hospital, Taoyuan, Taiwan; 2 College of Medicine, Chang Gung University, Taoyuan, Taiwan; 3 Department of Pediatrics, Chiayi Chang-Gung Memorial Hospital, No. 6, W. Sec., Jiapu Rd., Puzi City, Chiayi County Taiwan

**Keywords:** Child maltreatment, Physical abuse, Unintentional injury

## Abstract

**Objectives:**

This study aimed to identify the predictors of neurologic outcomes and mortality in physically abused and unintentionally injured children admitted to intensive care units (ICUs).

**Methods:**

All maltreated children were admitted to pediatric, neurosurgical, and trauma ICUs between 2001 and 2019. Clinical factors, including age, sex, season of admission, identifying settings, injury severity score, etiologies, length of stay in the ICU, neurologic outcomes, and mortality, were analyzed and compared between the physically abused and unintentionally injured groups. Neurologic assessments were conducted using the Pediatric Cerebral Performance Category scale. The study was approved by the Institutional Review Board of Chang Gung Memorial Hospital and the Ethics Committee waived the requirement for informed consent because of the anonymized nature of the data.

**Results:**

A total of 2481 children were investigated; of them, there were 480 (19.3%) victims admitted to the ICUs, including 156 physically abused and 324 unintentionally injured. Age, history of prematurity, clinical outcomes, head injury, neurosurgical interventions, clinical manifestations, brain computed tomography findings, and laboratory findings significantly differed between them (all *p* < 0.05). Traumatic brain injury was the major etiology for admission to the ICU. The incidence of abusive head trauma was 87.1% among the physically abused group. Only 46 (29.4%) and 268 (82.7%) cases achieved favorable neurologic outcomes in the physically abused and unintentionally injured groups, respectively. Shock within 24 h, spontaneous hypothermia (body temperature, < 35 °C), and post-traumatic seizure were strongly associated with poor neurologic outcomes and mortality in both groups.

**Conclusions:**

Initial presentation with shock, spontaneous hypothermia at ICU admission, and post-traumatic seizure were associated with poor neurologic outcomes and mortality in physically abused and unintentionally injured children.

**Supplementary Information:**

The online version contains supplementary material available at 10.1186/s40001-023-01430-x.

## Introduction

Child maltreatment and the associated morbidity and mortality are a critical and complex public health issue worldwide. It is generally recognized as physical abuse, neglect, emotional abuse, and sexual abuse. Physical abuse and neglect are the two most common types of child maltreatment and are considered serious causes of child mortality [1]. These cases are defined as events that occur as a result of injury caused by inflicted or careless consequences. According to the World Health Organization, physical abuse yields the highest mortality rate among the types of child maltreatment [2, 3]. Studies reveal that about a quarter and one-sixth of children had suffered physically abuse and neglect respectively in their childhood worldwide [3]. In other studies, the mortality rate was reported to be approximately 9% in physically abused victims and 2% in neglected victims [4–6]. Cases of neglect may include traffic accidents, falls, near drowning, burns, substance poisoning, and asphyxia, which are considered unintentional injuries [3]. Victims of physical abuse were reported to have higher proportions of injuries in the head, thoracic region, and skin than those of neglect [1, 2]. Physical abuse involving the head is called abusive head trauma (AHT); it is the most critical form of child maltreatment with a mortality rate of 20% and permanent neurologic damage rate of up to 50% [6, 7].

Because of the poor prognosis associated with child maltreatment, medical or surgical treatments should be performed immediately in critically ill victims, and the potential prognostic factors should be identified to avoid poor clinical outcomes. However, to date, only a few studies have focused on physically abused and unintentionally injured children admitted to the intensive care unit (ICU) [1, 2, 4]. Therefore, this study aimed to evaluate victims of physical abuse and unintentional injury admitted to the ICU and to analyze the related predictors of clinical outcomes.

## Materials and methods

### Study population

From 2001 to 2019, we retrospectively evaluated physically abused and unintentionally injured victims aged 1 month to 18 years admitted to the pediatric, neurosurgical, and trauma ICUs from the emergency department (ED), outpatient department (OPD), and wards. A total of 2481 children reported from the social welfare reporting system were included in this study. The study was approved by the Institutional Review Board of Chang Gung Memorial Hospital (reference number: 104-8307B). All procedures were performed in accordance with the relevant guidelines and regulations. Data were collected, reviewed, de-identified, and anonymized before analysis, and the Ethics Committee waived the requirement for informed consent because of the anonymized nature of the data and scientific purpose of the study.

### Study setting and patient selection

Chang Gung Memorial Hospital is a tertiary medical center that receives cases transferred from local clinics, regional hospitals, and social and political institutions. The pediatric ICU has 29 beds, wherein patients aged 1 month to 18 years are hospitalized. Meanwhile, the neurosurgical and trauma ICUs have 30 and 28 beds, respectively, wherein patients with severe brain injury or physical trauma requiring medical or surgical treatments are managed. In the study, all maltreated children admitted to the ICUs were divided into two major groups based on the etiologies, i.e., physical abuse and unintentional injury. Physical abuse is defined as acts of commission by a caregiver that cause actual physical harm or have the potential for harm. Neglect is defined as a failure by a child’s caregiver to meet the physical, emotional, educational, or medical needs of the child. Unintentional injury is a part of neglect that causes physical damage (traffic accidents, falls, near drowning, burns, substance poisoning, and asphyxia). All victims of physical abuse and unintentional injury were evaluated by a child protection team.

The inclusion criteria for this study were: (1) age from 1 month to 18 years; (2) diagnosed with physical abuse or unintentional injury; (3) admitted to the ICUs. The exclusion criteria were: (1) those older than 18 years; (2) diagnosed with other forms of child maltreatment such as emotional or sexual abuse (3) not admitted to the ICUs; (4) patients with prior central nervous system diseases or developmental delay before the injury.

### Study protocol

Information related to the cases of physical abuse and unintentional injury was obtained from the social welfare reporting system and the medical chart records; this included age, sex, personal and family histories, types of abuse, types of injury, initial clinical presentations on admission, physical examinations, laboratory tests, imaging examinations, length of stay (LOS) in hospitals, LOS in the ICU, time of presentation (morning shift, night shift, or graveyard shift), time of report (8 a.m.–5 p.m., 5 p.m.–0 a.m., and 0 a.m.–8 a.m.), transferring unit (ED, OPD, wards, or other hospitals), morbidity, neurologic outcomes, and mortality rate. Admission to the ICU was considered to indicate an urgent and critical condition requiring emergency critical care and a high risk of mortality. The factors associated with both mortality and neurologic outcomes were analyzed between the physically abused and unintentionally injured groups. Moreover, the predictors of mortality were further analyzed in each group using a logistic regression analysis.

Shock is defined as a systolic blood pressure (SBP) that is less than the 5th percentile for age. The age-related criteria for shock reported by the Pediatric Advanced Life Support are listed below: term neonates (0–28 days): < 60 mmHg; infant (1–12 months): < 70 mmHg; children (1–10 years old): < (70 mmHg + [2 × age in years]); children (≧ 10 years old): < 90 mmHg [8, 9].

### Outcome variables

The in-hospital mortality and neurologic outcomes of the survivors were the main outcome variables of this study. Meanwhile, the general functional status at discharge was the secondary outcome variable, assessed using the Pediatric Cerebral Performance Category (PCPC) scale (Additional file 2: Table S1). Neurologic outcome at discharge was evaluated by the pediatric neurologist using the system of Pediatric Cerebral Performance Category (PCPC) Scale. Outcome scores were divided into 2 levels as 1. favorable neurologic outcome (PCPC scale score, ≤ 2) and 2. poor neurologic outcome (PCPC scale score, ≥ 3). A survival analysis was conducted to compare the different survival rates between the physically abused and unintentionally injured groups according to the period of critical care in the ICU.

### Statistical analysis

The chi-square test, Fisher’s exact test, Student’s t-test, the Mann–Whitney U test, and a multivariate logistic regression analysis were used, where appropriate. In the descriptive analysis, values were presented as means ± standard deviations. Differences between the groups were presented as 95% confidence intervals (CIs). The chi-square test or Fisher’s exact test was used to compare dichotomous variables and the Mann–Whitney U test to compare continuous variables between the groups. The predicted probabilities of mortality and 95% CIs were calculated using a logistic regression model. The log-rank test and Kaplan–Meier survival curves were applied to compare survival differences. A Cox proportional hazard model was used to test the effect of independent variables on hazards. Statistical significance was set at *p*-values of < 0.05, and all statistical analyses were conducted using the IBM SPSS Statistics software (version 22.0; SPSS Inc., Chicago, IL, USA).

## Results

### Demographics

During the 19-year study period, 2481 cases of child abuse were reported via the social welfare reporting system; there were 480 (19.3%) patients admitted to the ICU for critical care, including 304 (63.3%) boys and 176 (36.7%) girls (Additional file 1: Fig. S1). Among them, 156 (32.5%) were physically abused, and 324 (67.5%) were unintentionally injured. The demographics between the two groups are shown in Additional file 3: Table S2. Age, history of prematurity, clinical outcomes, head injury, neurosurgical interventions, clinical manifestations, brain computed tomography (CT) findings, and laboratory findings significantly differed between them (all *p* < 0.05). Traumatic brain injury (TBI) was the major etiology for admission to the ICU, including AHT and accidental TBI. The incidence of AHT was 87.1% in the physically abused group, and that of accidental TBI was 57.4% in the unintentionally injured group (*p* < 0.01). In terms of brain CT findings, subdural hemorrhage (SDH) and subarachnoid hemorrhage were more common in the physically abused group, while epidural hemorrhage was more predominant in the unintentionally injured group (both *p* < 0.05). Moreover, neurosurgery was more commonly performed in the physically abused group than in the unintentionally injured group (53.2% vs. 18.5%; *p* < 0.05). In terms of blood laboratory findings, the physically abused group had lower hemoglobin level, platelet count, and serum sodium level but higher blood pH and serum potassium level than the unintentionally injured group (all p < 0.05). In terms of clinical presentation, shock, retinal hemorrhage, and convulsions were more common in the physically abused group than in the unintentionally injured group (all *p* < 0.05). In terms of clinical outcomes, the LOS in both the hospital and ICU was longer in the physically abused group than in the unintentionally injured group (both *p* < 0.05). In addition, the proportion of victims with worse neurologic outcomes (PCPC scale score, ≥ 3) was higher in the physically abused group than in the unintentionally injured group (*p* < 0.05).

### Factors associate with poor neurologic outcomes

The results of the comparison of the favorable and poor neurologic outcomes between the two groups are listed in Table 1. A total of 110 (70.6%) and 56 (17.3%) children had poor neurologic outcomes in the physically abused group and unintentionally injured group, respectively. Moreover, the children with poor neurologic prognosis had significantly higher rates of shock, head injury, and convulsions and lower initial Glasgow Coma Scale (GCS) score and body temperature in both groups (all *p* < 0.05). In the multivariate logistic regression analysis, shock within 24 h of ICU admission and initial presentation of convulsions were both found to be associated with poor neurologic outcomes in both groups (both *p* < 0.05) (Table 2). However, retinal hemorrhage was associated with poor neurologic outcomes in only the physically abused group and spontaneous hypothermia (body temperature, < 35 °C) in only the unintentionally injured group. Among the related significant clinical factors, shock within 24 h of ICU admission yielded the highest odds ratio for poor neurologic outcomes in both groups.

**Table 1:** Correlation between prognosis and characteristics in the abused and neglected group

Prognosis	Physical abuse (n = 156)			Unintentional injury (n = 324)		
	Favorable	Poor	*P* value	Favorable	Poor	*P* value
Patients	46 (29.4)	110 (70.6)		268 (82.7)	56 (17.3)	
Age (years), median (IQR)	0.6 (0.3–1.5)	0.5 (0.2–0.7)	0.8	3 (1–5)	3.5 (1–7.2)	0.28
Gender			0.92			0.84
Male	28 (60.8)	66 (60)		176 (65.6)	36 (64.2)	
Female	18 (39.2)	44 (40)		92 (34.4)	20 (35.8)	
LOS in ICU	9.1 ± 7.2	19.8 ± 22.6	< 0.01^*^	5.9 ± 5.1	16.9 ± 18.7	< 0.01^*^
Initial presentation						
Initial GCS	14 (11–15)	10 (6–13)	0.029	15 (15–15)	8 (6–9)	< 0.01^*^
Shock	1 (2.1)	31 (28.2)	< 0.01*	3 (1.1)	31 (55.3)	< 0.01^*^
Head injury	31 (67.3)	105 (95.4)	< 0.01*	136 (50.7)	50 (89.2)	< 0.01^*^
Brain CT findings						
SDH	25 (54.3)	87 (79)	0.003	50 (18.7)	14 (25)	0.28
EDH	1 (2.1)	1 (0.9)	1	42 (38.2)	7 (12.5)	0.54
SAH	11 (23.9)	29 (26.4)	0.75	20 (7.5)	2 (3.6)	0.26
ICH	0	14 (12.7)	0.026	15 (5.6)	4 (7.1)	0.66
HIE	2 (4.2)	23 (20.9)	0.01	10 (3.7)	30 (53.6)	< 0.001
Neurosurgery	20 (43.4)	63 (57.2)	0.07	45 (16.7)	15 (26.7)	0.07
Clinical presentations						
Fracture	15 (32.6)	34 (30.9)	0.76	89 (33.2)	27 (48.2)	0.03^*^
Convulsions	14 (30.4)	77 (70)	< 0.01^*^	24 (8.9)	12 (21.4)	0.01^*^
Retinal hemorrhage	18 (39.1)	87 (79.1)	< 0.01^*^	4 (1.4)	3 (5.3)	0.2
Body temperature	36.7 ± 0.7	35.7 ± 2.3	< 0.01^*^	36.7 ± 1	34.9 ± 2.8	0.018^*^
Laboratory findings						
Blood pH	7.42 ± 0.07	7.33 ± 0.16	0.05	7.36 ± 0.1	7.14 ± 0.23	< 0.01^*^
Sugar (mg/dL)	121 ± 52	172 ± 102	0.01^*^	249 ± 137	168 ± 108	< 0.01^*^
WBC (/uL)	12576 ± 3698	15582 ± 7414	0.01^*^	15816 ± 7280	18195 ± 9468	0.12
Hb (g/dL)	10.6 ± 1.9	9.5 ± 2.1	0.03^*^	11.8 ± 1.6	11.5 ± 2.7	0.44
Platelet (/uL)	395 ± 134	362 ± 159	0.38	322 ± 103	291 ± 128	0.08
PT (sec)	13 ± 1	16 ± 13	0.26	13 ± 3	22 ± 19	< 0.01^*^
aPTT (sec)	31 ± 7	35 ± 15	0.36	29 ± 5	42 ± 23	< 0.01^*^
Calcium (mg/dL)	9.6 ± 0.7	8.9 ± 0.9	< 0.01^*^	9.2 ± 0.8	8.4 ± 1	< 0.01^*^
Phosphate (mg/dL)	4.9 ± 1.2	5.5 ± 1.7	0.26	5.5 ± 1.6	6.9 ± 2.7	0.02^*^
Creatinine (mg/dL)	0.5 ± 0.4	0.5 ± 0.2	0.16	0.4 ± 0.2	0.8 ± 0.4	0.02^*^

ICU: intensive care unit; LOS: length of stay; WBC: white blood count; Hb: Hemoglobin; PT: prothrombin time; aPTT: activated partial thromboplastin time; BUN: blood urea nitrogen

(*): %;^*^: p < 0.05 statistic significant

**Table 2:** Logistic regression analysis of the related risk factors for poor neurologic outcomes

Group	Physical abuse		Unintentional injury	
	Odds ratios (95% CI)	p-value	Odds ratios (95% CI)	p-value
Univariate logistic regression				
Shock	17.6 (2.3–133.7)	0.005^*^	109.5 (31.2–383.8)	< 0.001^*^
Initial GCS	0.81 (0.65–0.99)	0.044^*^	0.53 (0.25–0.96)	0.03^*^
Head injury	10.1 (3.4–30.2)	< 0.001^*^	8.1 (3.4–19.5)	< 0.001^*^
SDH	3.1 (1.5–6.6)	0.002^*^	1.45 (0.74–2.86)	0.28
HIE	2.3 (1.9–8.9)	< 0.001^*^	29.7 (13.1–67.7)	< 0.001^*^
Convulsions	5.3 (2.5–11.2)	< 0.001^*^	2.7 (1.3–5.9)	0.009^*^
Retinal hemorrhage	5.8 (2.8–12.4)	< 0.001^*^	3.75 (0.74–18.95)	0.11
Spontaneous hypothermia	17 (2.2–130.5)	0.006^*^	15.1 (7.3–31.3)	< 0.001
Hb (g/dL)	0.76 (0.59–0.98)	0.035^*^	0.93 (0.78–1.11)	0.44
Calcium(mg/dL)	0.17 (0.05–0.53)	0.002^*^	0.35 (0.19–0.66)	0.001^*^
Fracture	1.05 (0.38–2.91)	0.92	2.8 (0.1–0.65)	< 0.001^*^
Sugar (mg/dL)	1.008 (0.99–1.02)	0.083	54.4 (9.8–301.5)	< 0.001^*^
PT (sec)	1.18 (0.9–1.55)	0.22	1.4 (1.1–1.6)	< 0.001^*^
aPTT (sec)	1.02 (0.96–1.09)	0.36	1.08 (1.03–1.13)	< 0.001^*^
Phosphate (mg/dL)	1.29 (0.85–1.97)	0.22	1.41 (1.09–1.83)	0.009^*^
Creatinine (mg/dL)	0.46 (0.12–1.79)	0.26	1.002 (1–1.003)	0.046^*^
Multivariate logistic regression				
Shock	21.4 (2.5–181)	0.005^*^	120.4 (13.5–1073)	< 0.001^*^
Convulsions	7.2 (2.7–18.9)	0.001^*^	5.1 (1.7–15)	0.003^*^
Spontaneous hypothermia	5.29 (0.55–51.1)	0.149	4.2 (1.4–12.4)	0.01^*^
Retinal hemorrhage	3.9 (1.5–10.5)	0.005^*^	5.34 (0.16–17.24)	0.35

CI: confidence interval; PT: prothrombin time; aPTT: activated partial thromboplastin time;

^*^: p < 0.05 statistic significant

### Mortality

#### Causes of mortality

In the physically abused group, 19 (95%) patients expired because of AHT. The other patient expired because of intentional strangulation by the father. In the unintentionally injured group, 10 (37%) expired owing to drowning; 6 (22.2%), suffocation; 3 (11.1%), falls; 3 (11.1%), carbon monoxide intoxication; 3 (11.1%), sudden infant death syndrome; and 2 (11.1%), road traffic accidents.

#### Factors associated with mortality

**Fig. 1:**
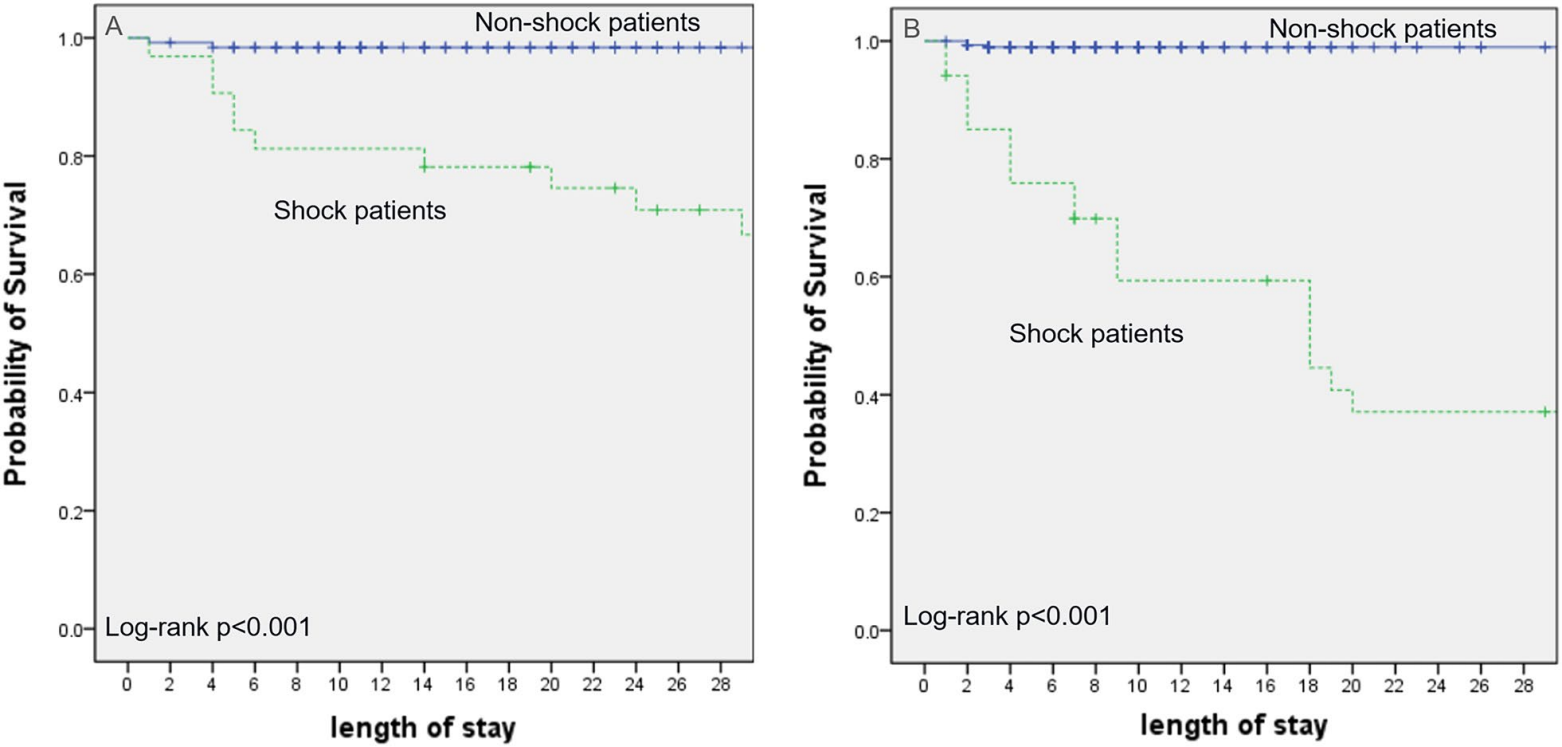
Kaplan–Meier Plot of the Probability of Survival Until day 28 between shock and non-shock patients in both groups. **A** Physical abuse **B** Unintentional injury

**Fig. 2:**
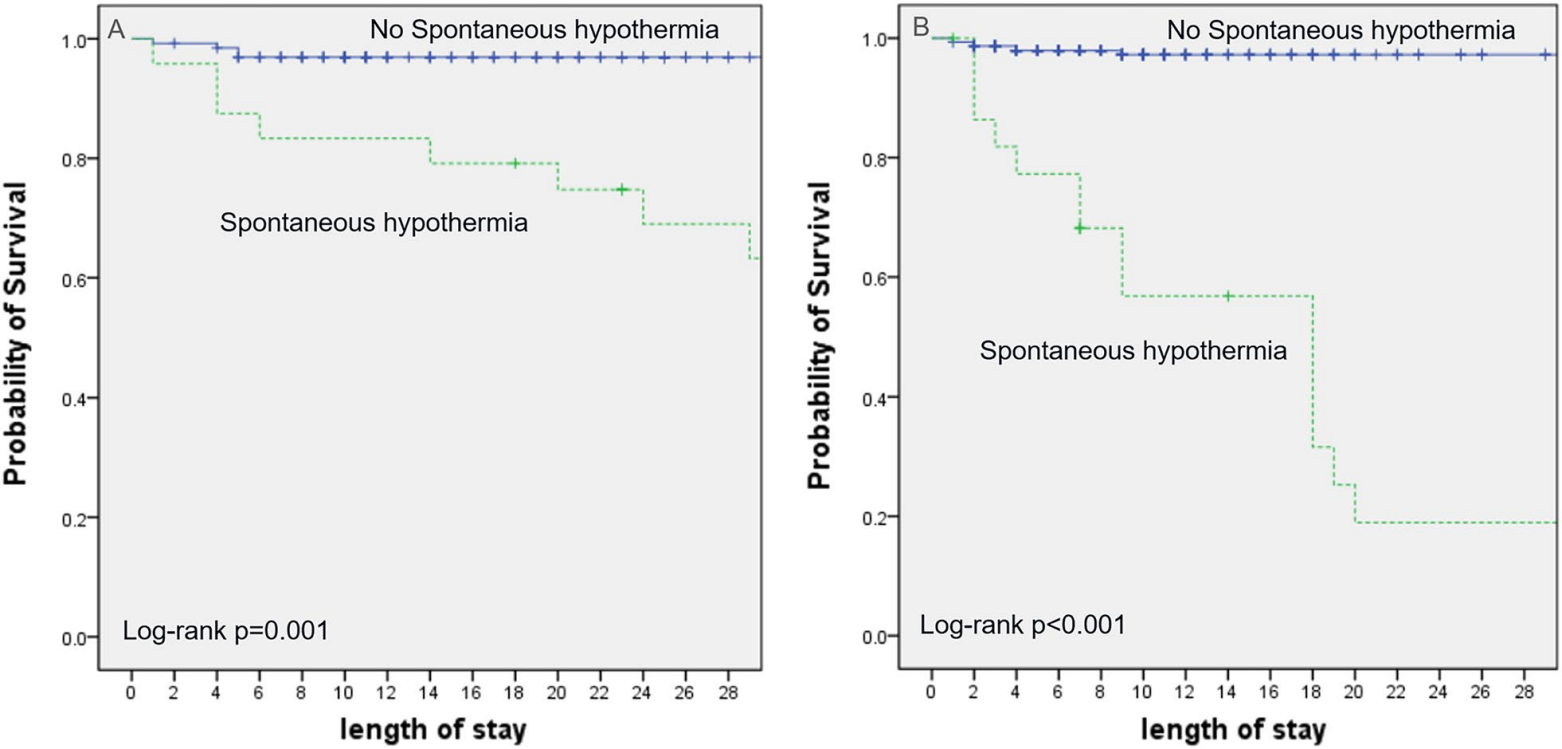
Kaplan–Meier Plot of the Probability of Survival Until day 28 between with and without spontaneous hypothermia patients in both groups. **A** Physical abuse **B** Unintentional injury

The overall mortality rate among the study population was 9.8%; specifically, the mortality rate was 12.8% in the physically abused group and 8.3% in the unintentionally injured group. Table 3 shows the Cox proportional hazards for predicting 28-day mortality. Shock within 24 h of ICU admission was the strongest predictor of 28-day mortality, followed by spontaneous hypothermia in both groups. The rate of poor neurologic prognosis (PCPC scale score, ≥ 3) was also significantly higher in the children with spontaneous hypothermia on admission to the ICU (93.5%) than in those without (28.1%, *p* < 0.05). The Kaplan–Meier survival curves in the two groups are shown in Figs. 1 and 2. The occurrence of initial shock within 24 h of ICU admission was analyzed using the Kaplan–Meier survival curves in the physically abused (Fig. 1A) and unintentionally injured groups (Fig. 1B). The proportion of victims who initially presented with shock and then expired was higher, and those who initially presented with shock expired earlier than did those who did not in both groups. Meanwhile, the proportion of victims who initially presented with spontaneous hypothermia and then expired was higher, and those who initially presented with spontaneous hypothermia expired earlier than did those who did not in the physically abused (Fig. 2A) and unintentionally injured groups (Fig. 2B).

**Table 3:** Risk of Death Within 28 Days in the Two groups

Group	Physical abuse		Unintentional injury	
	Hazard Ratio (95% CI)	p-value	Hazard Ratio (95% CI)	p-value
Shock	9.1 (2.2–37.3)	< 0.001^*^	16.9 (4.9–58.5)	< 0.001^*^
Convulsions	3 (0.7–8)	0.06	1.1 (0.2–5.1)	0.94
Spontaneous hypothermia	4.9 (1.8–13.3)	0.002^*^	2.9 (1.2–7.1)	0.02^*^
Retinal hemorrhage	1.03 (0.3–3.3)	0.996	0.41 (0.05–3.54)	0.42

CI: confidence interval

^*^: p < 0.05 statistic significant

Approximately 80% of mortality caused by AHT may be survived with earlier interventions [10]. Primary clinicians should be highly alert to recognize the possibility of child maltreatment. However, timely diagnosis of AHT is difficult because the initial presentations often involve nonspecific complaints, such as difficulty feeding and breathing, poor activity, apnea, convulsions, or drowsiness; this consequently causes confusion on the diagnostic process among doctors [11]. Differentiating between AHT and accidental TBI may be equivocal; this is especially true, as histories provided are usually fictitious to conceal abuse.

## Discussion

This study is the first to analyze the risk factors associated with the clinical prognosis of maltreated children admitted to the ICU. Herein, we included and investigated up to 480 victims admitted to the ICU within a relatively long study period (19 years). The ICU admission rate was 19.3% in the total study population of 2,481 children, which indicates that 1 in every 5 cases required critical care owing to child maltreatment. The mortality rate was 12.8% in the physically abused group and 8.3% in the unintentionally injured group, which was both higher than previous rates [5, 6]. We think that this may be attributed to the rarely reported cases from the medical and social systems in the past. Only very critically ill victims were admitted to the ICU and reported in the past.

In our study, we identified that initial presentation with shock within 24 h was the strongest predictor of poor prognosis in both groups. The major type of shock observed was neurogenic shock, mainly caused by TBI, which accounted for approximately 51% of the mortality herein. TBI is a leading cause of morbidity and mortality in child maltreatment cases worldwide, and previous studies have demonstrated several risk factors associated with poor outcomes in children with TBI, including low GCS score, post-traumatic seizure, retinal hemorrhage, young age, and SDH [8, 12–16]. However, in our study, we found that shock, head injury, convulsions, retinal hemorrhage, lower initial GCS score, and lower body temperature were all risk factors of poor neurologic outcomes. Severe brain swelling and mass effect caused by TBI may lead to neurogenic shock. Shock may cause ischemic injuries and reperfusion impairment, which may increase the rates of cell death and multi-organ failure, resulting in high mortality rates [17–20]. In our study, the children with shock expired earlier and easily and had poor neurologic outcomes. Therefore, the occurrence of shock within 24 h of ICU admission may indicate a high mortality and a poor neurologic outcome in physically abused or unintentionally injured children. Hence, closely monitoring the early signs of shock, such as tachycardia, poor perfusion, and low blood pressure, and emergent fluid resuscitation with vasopressors could be important for children with TBI.

The second strongest risk factor associated with mortality in our study was spontaneous hypothermia following injuries. Previous studies have demonstrated that spontaneous hypothermia was a risk factor for mortality in adult patients with severe trauma and hemorrhage and identified it as a new risk factor after cardiac arrest in adults [21–23]. Under normal conditions, body temperature is regulated by the hypothalamus. Spontaneous hypothermia after severe injuries may indicate a damage to mechanism of thermoregulation by the cerebrum resulting from ischemia and reperfusion damage. Therefore, spontaneous hypothermia may be translated as a sign of cerebral injury [23]. In addition, hypothermia may disrupt systemic homeostasis, deplete energy stores, and finally cause multi-organ failure [22]. This is the first study to analyze the impact of body temperature changes on ICU admission in abused children. In physically abused and unintentionally injured children, critical conditions with spontaneous hypothermia may indicate a high mortality rate. Moreover, spontaneous hypothermia may also serve as a predictor of poor neurologic prognosis in children with unintentional injuries. When shock and spontaneous hypothermia occur in physically abused and unintentionally injured children, the harmful changes become additive, and a higher mortality rate is ensued.

Herein, we also found that convulsion was not only a common post-trauma complication but also a risk factor for poor neurologic outcomes in both the physically abused and unintentionally injured groups. The correlation between convulsion and poor neurologic outcomes may be attributed to the consequent increases in metabolic demands, intracranial pressure, and release of neurotransmitters arising from secondary brain injury [14, 24]. Therefore, early detection using continuous electroencephalographic monitoring and antiepileptic drug administration could be recommended for pediatric TBI to avoid progression of poor neurologic outcomes [25, 26]. Although traumatic causes may result in a pattern of retinal hemorrhage, this type of hemorrhage is still an important manifestation of AHT and frequently observed particularly during dilated eye examination in approximately 85% of children with AHT. In our study, retinal hemorrhage was found to be a risk factor for poor neurologic outcomes among the physically abused children but not among the unintentionally injured children. The presence of retinal hemorrhage may have a nonspecific pattern in children with AHT; however, once retinal hemorrhages are noted in children with AHT, poor neurologic outcomes may be expected. Therefore, primary clinicians should pay more attention to performing dilated eye examinations in children suspected to have experienced child maltreatment because the presence of retinal hemorrhage may not only identify the existence of child abuse but also indicate the possibility of poor neurologic outcomes.

Several limitations were noted in the current study. First, the study was reviewed retrospectively at a single center, and therefore, there were risks of missing data and information bias. The result should be used with caution. Second, the study was carried out over 18 years. The evolution of therapeutic strategies over that time span may have impacted the outcomes. However, the therapeutic strategy for maltreated children have not changed fundamentally which may have reduced the risk of bias.

## Conclusions

Initial presentation with shock, spontaneous hypothermia at ICU admission, and post-traumatic seizure were associated with poor neurologic outcomes and mortality in physically abused and unintentionally injured children. Further prospective studies focusing on treating these risk factors to improve outcomes are warranted.

### Supplementary Information

**Additional file 1: Figure S1.** Cases of child maltreatment since 2001 to 2019.

**Additional file 2: Table S1.** Pediatric Cerebral Performance Category Scale (PCPC).

**Additional file 3: Table S2.** Demographics of Forensic Cases admitted to the ICUs.

## Data Availability

The datasets used and analyzed during the current study are available from the corresponding author on reasonable request.
