# Supplementary figures and images for: Predictors of neurologic outcomes and mortality in physically abused and unintentionally injured children: a retrospective observation study
Source: Eur J Med Res. 2023 Oct 17;28:441. doi: 10.1186/s40001-023-01430-x (PMC10580634; doi:10.1186/s40001-023-01430-x)

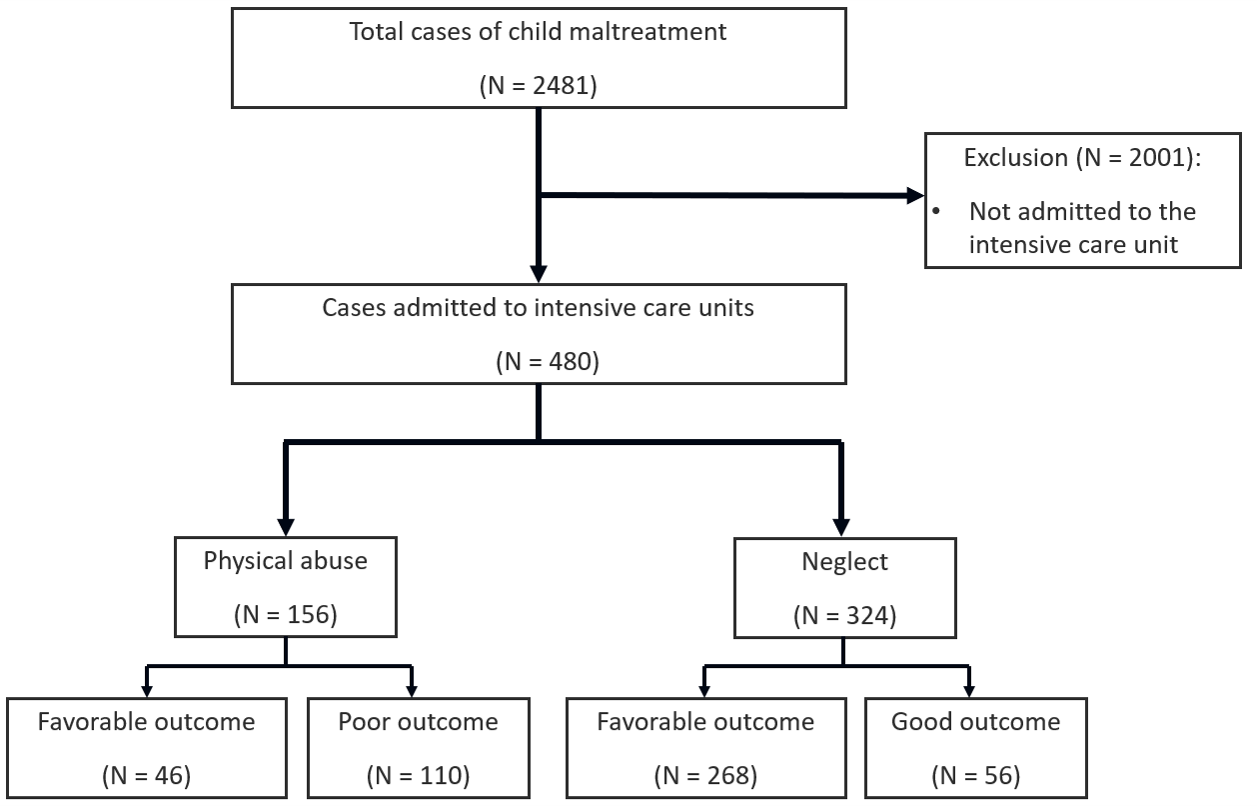

Supplement: Supplementary file 1 — Additional file 1: Figure S1. Cases of child maltreatment since 2001 to 2019. [file 40001_2023_1430_MOESM1_ESM.tif]
