# Supplementary material for: Predictors of neurologic outcomes and mortality in physically abused and unintentionally injured children: a retrospective observation study
Source: Eur J Med Res. 2023 Oct 17;28:441. doi: 10.1186/s40001-023-01430-x (PMC10580634; doi:10.1186/s40001-023-01430-x)
Supplement: Supplementary file 2 — Additional file 2: Table S1. Pediatric Cerebral Performance Category Scale (PCPC). [file 40001_2023_1430_MOESM2_ESM.doc]

Additional Table 1. Pediatric Cerebral Performance Category Scale (PCPC).

| **Pediatric Cerebral Performance Category Scale (PCPC)** | | |
| --- | --- | --- |
| **Normal**  **(score = 1)** | *Age-appropriate level of functioning  *In preschool-aged children, appropriate development  *In school-aged children, attendance in regular classes | |
| **Mild Disability**  **(score = 2)** | *Can interact at an age-appropriate level  *Minor neurologic disease that is controlled and does not interfere with daily functioning (eg, seizure disorder)  *In preschool-aged children, possibly minor developmental delays, but with > 75% of all daily living developmental milestones above the 10th percentile  *In school-aged children, attendance in regular school but in a grade that is not appropriate for age or in the appropriate grade but failing because of cognitive difficulties | |
| **Moderate Disability**  **(score = 3)** | *Below age-appropriate functioning  *Neurologic disease that is not controlled and severely limits activities  *In preschool-aged children, most daily living developmental milestones below the 10th percentile  *In school-aged children, can do ADLs but attend special classes because of cognitive difficulties or a learning deficit | |
| **Severe Disability**  **(score = 4)** | *In preschool-aged children, ADLs milestones below the 10th percentile and excessive dependence on others for activities of daily living  *In school-aged children, possibly severe impairment that prevents school attendance and dependence on others for ADLs  *In preschool-aged and school-aged children, possibly abnormal motor movements, including non-purposeful, decorticate, or decerebrate responses to pain | |
| **Coma/vegetative**  **(score = 5)** | *Unaware, even if awake in appearance, without interaction with environment  *Cerebral unresponsiveness and no evidence of cortical function (i.e. not aroused by verbal stimuli)  *Possibility of some reflective response, spontaneous eye-opening and sleep-wake cycles | |
| **Brain death**  **(score = 6)** | | Apnea, areflexia, and/or electroencephalographic silence |

Fiser DH. Assessing the outcome of pediatric intensive care.  *J Pediatr* 121(1):68-74, 1992. doi:10.1016/s0022-3476(05)82544-2
