# Supplementary material for: Predictors of neurologic outcomes and mortality in physically abused and unintentionally injured children: a retrospective observation study
Source: Eur J Med Res. 2023 Oct 17;28:441. doi: 10.1186/s40001-023-01430-x (PMC10580634; doi:10.1186/s40001-023-01430-x)
Supplement: Supplementary file 3 — Additional file 3: Table S2. Demographics of Forensic Cases admitted to the ICUs. [file 40001_2023_1430_MOESM3_ESM.doc]

| Additional Table 2. Demographics of Forensic Cases admitted to the ICUs | | | |
| --- | --- | --- | --- |
| Variables | Physical abuse | Unintentional injury | p-value |
| Patient number | 156 (32.5) | 324 (67.5) |  |
| **Age** (years), median (IQR) | 0.5 (0.2-0.83) | 3 (1-6) | <0.01^＊^ |
| **Gender** |  |  | 0.19 |
| Male | 92 (58.9) | 212 (65.4) |  |
| Female | 64 (41.1) | 112 (34.6) |  |
| **Personal History** |  |  |  |
| Prematurity | 20 (12.8) | 17 (5.2) | <0.01^＊^ |
| Chronic medical condition | 18 (11.5) | 16 (4.9) | <0.01^＊^ |
| **Identifying settings** |  |  | 0.32 |
| Emergent department | 70 (44.8) | 164 (50.6) |  |
| Outpatient department | 1 (0.6) | 2 (0.6) |  |
| Transfer | 88 (54.6) | 159 (48.8) |  |
| **Time of report** |  |  | 0.11 |
| 8:00~17:00 | 58 (37.1) | 93 (28.7) |  |
| 17:00~24:00 | 54 (34.6) | 152 (46.9) |  |
| 0:00~8:00 | 47 (28.3) | 80 (24.4) |  |
| **Outcomes** | | | |
| Length of stay (days) | 24 ± 24.1 | 11.5 ± 11.8 | <0.01^＊^ |
| ICU LOS (days) | 16.6 ± 20 | 7.8 ± 9.9 | <0.01^＊^ |
| Mortality | 20 (12.8) | 27 (8.3) | 0.13 |
| PCPC scale |  |  | <0.01^＊^ |
| Favorable prognosis | 46 (29.4) | 268 (82.7) |  |
| Poor prognosis | 110 (70.6) | 56 (17.3) |  |
| **Initial manifestation** |  |  |  |
| Initial GCS, median (IQR) | 12 (7-14) | 14 (10-15) | <0.01^＊^ |
| Head injuries | 136 (87.1) | 186 (57.4) | <0.01^＊^ |
| **Brain CT findings** |  |  |  |
| SDH | 112 (71.7) | 64 (19.7) | <0.01^＊^ |
| EDH | 2 (1.2) | 49 (15.1) | <0.01^＊^ |
| SAH | 40 (25.6) | 22 (6.7) | <0.01^＊^ |
| ICH | 14 (8.9) | 19 (5.8) | 0.22 |
| HIE | 25 (16) | 40 (12.3) | 0.3 |
| Neurosurgical interventions | 83 (53.2) | 60 (18.5) | <0.01^＊^ |
| **Clinical presentations** |  |  |  |
| Convulsions | 91 (58.3) | 36 (11.1) | <0.01^＊^ |
| Shock | 32 (20.5) | 34 (10.4) | <0.01^＊^ |
| Retinal hemorrhage | 105 (67.3) | 7 (2.1) | <0.01^＊^ |
| Fracture | 52 (33.3) | 117 (36.1) | 0.48 |
| Body temperature | 35.9 ± 1.9 | 36.3 ± 1.6 | 0.06 |
| **Laboratory findings** |  |  |  |
| Blood pH | 7.35 ± 0.15 | 7.29 ± 0.19 | 0.03^＊^ |
| Sugar(mg/dL) | 164.3 ± 96.8 | 181.1 ± 116.7 | 0.30 |
| WBC(/uL) | 14944 ± 6893 | 16238 ± 7746 | 0.15 |
| Hb (g/dL) | 9.7 ± 2.1 | 11.7 ± 1.8 | <0.01^＊^ |
| Platelet (/uL) | 369 ± 154 | 317 ± 108 | <0.01^＊^ |
| PT(sec) | 16 ± 12 | 15 ± 10 | 0.52 |
| aPTT (sec) | 33.5 ± 12.7 | 31.9 ± 13.3 | 0.35 |
| Sodium (mEq/L) | 137 ± 5 | 138 ± 3 | 0.016^＊^ |
| Potassium (mEq/L) | 4.6 ± 1.3 | 4.1 ± 0.9 | <0.01^＊^ |
| Chloride (mEq/L) | 105 ± 6 | 106 ± 4 | 0.22 |
| Calcium (mg/dL) | 9 ± 0.9 | 9 ± 1 | 0.69 |
| Phosphate(mg/dL) | 5.4 ± 1.6 | 6 ± 2.1 | 0.06 |
| Magnesium(mEq/L) | 2 ± 0.3 | 2 ± 0.5 | 0.74 |
| BUN(mg/dL) | 10 ± 16 | 10.9 ± 4 | 0.81 |
| Creatinine(mg/dL) | 0.37 ± 0.44 | 0.43 ± 0.24 | 0.12 |

(＊) : % ;^＊^ : p < 0.05 statistic significant; ICU = intensive care unit; LOS = length of stay; PCPC = Pediatric Cerebral Performance Category; SDH = subdural hemorrhage ; EDH = epidural hemorrhage; SAH = subarachnoid hemorrhage; IVH = intraventricular hemorrhage; CPR = cardiopulmonary resuscitation; WBC : white blood count; Hb : Hemoglobin; PT : prothrombin time; aPTT : activated partial thromboplastin time; BUN : blood urea nitrogen.
